# Who is at risk for weight gain after weight‐gain associated treatment with antipsychotics, antidepressants, and mood stabilizers: A machine learning approach

**DOI:** 10.1111/acps.13684

**Published:** 2024-04-01

**Authors:** Julia Eder, Catherine Glocker, Barbara Barton, Elif Sarisik, David Popovic, Jana Lämmermann, Alexandra Knaf, Anja Beqiri‐Zagler, Katharina Engl, Leonie Rihs, Lisa Pfeiffer, Andrea Schmitt, Peter Falkai, Maria S. Simon, Richard Musil

**Affiliations:** ^1^ Department of Psychiatry and Psychotherapy LMU University Hospital, LMU Munich Germany; ^2^ Graduate Program “POKAL ‐ Predictors and Outcomes in Primary Care” (DFG‐GrK 2621) Munich Germany; ^3^ International Max Planck Research School for Translational Psychiatry (IMPRS‐TP) Munich Germany; ^4^ Max Planck Institute of Psychiatry Munich Germany; ^5^ Laboratory of Neuroscience (LIM27) Institute of Psychiatry, University of São Paulo São Paulo Brazil; ^6^ Oberberg Fachklinik Bad Tölz Bad Tölz Germany

**Keywords:** antidepressants, antipsychotics, mood‐stabilizer, predictor, weight gain

## Abstract

**Background:**

Weight gain is a common side effect in psychopharmacology; however, targeted therapeutic interventions and prevention strategies are currently absent in day‐to‐day clinical practice. To promote the development of such strategies, the identification of factors indicative of patients at risk is essential.

**Methods:**

In this study, we developed a transdiagnostic model using and comparing decision tree classifiers, logistic regression, XGboost, and a support vector machine to predict weight gain of ≥5% of body weight during the first 4 weeks of treatment with psychotropic drugs associated with weight gain in 103 psychiatric inpatients. We included established variables from the literature as well as an extended set with additional clinical variables and questionnaires.

**Results:**

Baseline BMI, premorbid BMI, and age are known risk factors and were confirmed by our models. Additionally, waist circumference has emerged as a new and significant risk factor. Eating behavior next to blood glucose were found as additional potential predictor that may underlie therapeutic interventions and could be used for preventive strategies in a cohort at risk for psychotropics induced weight gain (PIWG).

**Conclusion:**

Our models validate existing findings and further uncover previously unknown modifiable factors, such as eating behavior and blood glucose, which can be used as targets for preventive strategies. These findings underscore the imperative for continued research in this domain to establish effective preventive measures for individuals undergoing psychotropic drug treatments.

**Significant outcomes**

- It is possible to predict patients taking medication associated with PIWG that are at risk of gaining ≥5% of body weight during the period of 4 weeks.
- The machine learning (ML) models became more accurate, when eating behavior, blood glucose alongside other laboratory values, and waist circumference were considered.
- Research needs to focus more on modifiable risk factors for PIWG to establish and implement preventive programs for patients.

**Limitations**

- Prior medication history was not considered in the study, and combination therapy was allowed.
- High dropout rates among patients could have introduced attrition bias. Patients were lost to follow‐up mainly due to clinic discharge.
- The sample is not representative of outpatient settings, since only data of inpatients was considered.
- No external validation is available for the extended Model.

## 1 INTRODUCTION

Weight gain is a common side effect of antipsychotics, mood stabilizers and antidepressants.^1^, ^2^, ^3^ These medications are widely used, frequently administered as combination therapy,^4^ and often used off‐label.^5^, ^6^ Psychotropic induced weight gain (PIWG) can lead to obesity, diabetes and cardiovascular disease,^7^ contributing to a reduced life expectancy in patients with severe mental disorders.^8^ Furthermore, weight gain is associated with low drug adherence,^9^ which contributes to relapse.^10^ The exact mechanisms of PIWG have not been clearly understood yet, a multifactorial explanation can be assumed.^11^ PIWG can occur quickly within the first weeks of treatment and especially early weight gain was found to be predictive for further weight gain.^12^, ^13^ Vandenberghe et al.^13^ found that a weight gain of more than 5% after 4 weeks of treatment is predictive of further weight gain of ≥15% after 3 months and ≥ 20% after 12 months. Accordingly, it is recommended to assess the possibility of changing the medication or providing dietary advice together with increased physical activity if a weight gain of 7% after 6 weeks of treatment has occurred.^14^ These interventions lead to a significant but only small reduction in weight, without yielding long‐term effects for a relevant reduction in body weight.^15^

Adding medication to prevent additional weight gain or induce weight loss is becoming an increasingly widespread strategy.^16^ Drugs like metformin^17^ or liraglutide^18^ have shown efficacy, and metformin (first choice) or topiramate (second choice) are recommended in the German S3 Schizophrenia guidelines.^19^ Unfortunately, the success of these interventions is also limited, leading to combination therapy with possibly more adverse events.

Since losing weight is very difficult, especially for patients with mental disorders,^20^ a more effective strategy would be to identify those with a high risk for PIWG before the start of treatment and implement preventive strategies simultaneously with the administration of relevant psychotropics. So far, several risk factors for PIWG have been identified. These include non‐white ethnicity,^3^ female gender,^21^, ^22^ young age and early weight gain on the long term,^3^, ^23^ a high premorbid BMI, low baseline BMI, and suffering from a schizophrenia spectrum disorder,^23^ being a non‐smoker,^23^ and not suffering from an addiction.^24^ Regarding the new administration of PIWG‐associated antidepressants and antipsychotics, a low baseline BMI at baseline^13^, ^25^ and low triglyceride level^26^ were associated with a higher risk for accelerated weight gain. Harrison et al.^27^ found that expression and genetic data did not improve prediction for PIWG as opposed to only using clinical data in their regression and classification models.

Most of these predictors cannot be influenced by the patients, as factors as one's ethnicity, baseline BMI or family history cannot be changed. In cases where treatment with a “weight gain drug” is without alternative despite a high individual risk for PIWG, changeable risk factors would enable the patient change specific behaviors that are associated with PIWG. For example, antipsychotics seem to influence the perception of satiety,^28^ which might affect eating behavior resulting in an increased food intake. However, not every patient with an increased appetite experiences weight gain,^13^, ^29^ indicating that differences in coping with unwanted adverse effects (AE) like ravenous hunger might be more crucial than the AE itself. Other studies identified the lack of cognitive control in the presence of an increased appetite^30^ and changes in food craving^31^ to be predictive for PIWG. Coping strategies and eating behavior can be well addressed by behavioral therapy.

Considering these findings we wish to emphasize the importance of implementing preventive measures prior to weight gain, as the metabolic outcomes might be difficult to reverse once initiated.^16^ In this study, we aimed to develop a transdiagnostic model using logistic regression and decision tree classifiers to predict which patients are at risk to gain weight during the stay at a psychiatric ward, being prescribed medication with known increased risk for PIWG. Identifying patients at risk for PIWG is crucial for clinicians to intervene before adverse metabolic consequences occur, ultimately reducing the mortality gap between the psychiatric and general populations.^8^ So far to our knowledge this is the first study to deliberately investigating predictors with the potential to change. Understanding modifiable predictors allows for the development of interventions targeting these risk factors, potentially achieving greater success than current non‐specific lifestyle interventions.

## 2 METHODS

### 2.1 Study design

A naturalistic observational study was carried out at the Department of Psychiatry and Psychotherapy, University Hospital, LMU Munich between 2014 and 2017.

Due to the naturalistic setting data were gathered transdiagnostically and across drug classes, reflecting the clinical day‐to‐day life of the practitioner, who is confronted with clinical decisions like choosing the appropriate medication in non‐medication naïve patients.

The study was approved by the ethics committee of the LMU (protocol number 290.14), and all participants provided written informed consent prior to study inclusion. The principles of good clinical practice and the declaration of Helsinki and its subsequent revisions apply.

### 2.2 Patient population

One hundred sixty‐three inpatients aged 18–75 with all ICD‐10 F‐diagnoses, that started treatment with an antipsychotic, antidepressant, or mood stabilizer associated with weight gain according to Dent et al.^1^ and Bak et al.,^32^ were included in the Metabolism in Psychiatry (MiP) 1 study: amisulpride, clozapine, haloperidol, olanzapine, paliperidone, quetiapine, risperidone, citalopram, doxepin, amitriptyline, mirtazapine, paroxetine, lithium and valproate. Exclusion criteria were severe cognitive impairments or severe delusions with the inability to fill out questionnaires.

For external validation of the results, we used data generated by the MiP 3 study, registered in the German Clinical Trials Register (DRKS00025946) and approved by the local ethics committee (project no. 21‐0357). MiP 3 is an ongoing naturalistic prospective study recruiting patients with depression. All MiP 3 participants were diagnosed with unipolar depression, while patients with psychotic, bipolar disorders, and drug addictions were excluded. 38 MiP 3 patients fulfilled the inclusion criteria of the MIP 1 study and were taken into account. Since premorbid weight and premorbid BMI were not assessed in this data set, these values were subsequently requested by telephone, when participants had also agreed to participating in the Munich Mental Health Biobank.^33^

### 2.3 Procedure and measures

Patients were enrolled at the start of weight gain associated medication. Since Vandenberghe et al.^13^ showed that substantial weight gain after 4 weeks predicts long‐term weight gain and given that the average duration of inpatient stays in our hospital is also 4 weeks, we monitored our patients over this period.

Following variables at study inclusion were chosen by existing evidence of their predictive capability for PIWG:

Demographic variables age^23^ and gender^21^, ^22^ as well as patients' main diagnoses,^23^ possible addiction disorders,^24^ and smoking status^23^ were retrieved from the medical records. Body weight was measured to calculate the baseline BMI^13^, ^25^ using a calibrated scale. Waist circumference was taken according to the WHO.^34^ Premorbid BMI (prior to psychopathological processes)^23^ was assessed on the weight cycling questionnaire (Wallner‐Liebmann, personal communication). Laboratory values, such as triglyceride levels,^26^ were collected after an overnight fasting period.

We further added psychometric variables from our previous research that have the potential to serve as additional predictors and that could be influenced by lifestyle changes, thereby offering utility for preventive actions:

Eating behavior^35^ was assessed using the German version of the three‐factor‐eating questionnaire (FEV), where sum scores for the three subscales cognitive control (dietary restraint), disinhibition (loss of control), and the experienced feeling of hunger were calculated^36^, ^37^ as well as the Food‐Craving Inventory^38^ (German version “Fragebogen Food Craving” (FFC) by Dalkner et al., personal communication), a self‐rating measure consisting of four subscales that represent craving for carbohydrates, sweets, high‐fat food and fast‐food.

### 2.4 Statistics

Group comparisons were made between subjects who had gained at least 5% of their body weight and those who had not. Given the respective scale of measure, an independent T‐test, the Kruskal Wallis or the Chi‐Square and the exact Fisher test were used to compare the two cohorts at least 5% weight gain (*N* = 22) and less than 5% weight gain (*N* = 82). Further FDR correction was conducted if multiple statistical comparisons were performed.

Correlations between dependent and independent variables were assessed using Pearson's correlation coefficient. We used logistic regression to identify a linear model, which provides evidence concerning factors that are related to weight gain ≥5% in the MiP 1 sample. Pseudo R‐squared values,^39^ the *p*‐values, Odds ratios (ORs), and 95% confidence intervals (CIs) were calculated to assess the model.

A total of 103 patient data sets were utilized for training and testing the decision tree classifiers, as well as an XGBoost Classifier, an SVM (Support Vector Machine) Model, and logistic regression, with the aim of comparing their performances. Whether the ≥5% weight gain threshold was fulfilled or not resulted in being the target variable. Where 20% or more of the ‘literature search’ feature set, consisting of 10 columns of data, had missing values, the data set was removed for all models. The remaining missing values were imputed using K‐nearest neighbors (KNN) imputation.

Within the nested 5 × 5 cross‐validation framework, logistic regression, the XGBoost algorithm, and the SVM model were scaled using the Robust Scaler provided by scikit‐learn. The decision tree model was not scaled.

For external validation of the study results, data generated by the MiP 3 study was used. In this work we focus on the decision tree classifier for its clinical translatability, simplicity of implementation, and interpretability, providing valuable insights into complex contexts.^40^ In order to construct a decision tree, Scikit‐learn uses an optimized version of the CART algorithm.^41^ This algorithm builds binary trees that provide the greatest information gain per node.

**FIGURE 1:**
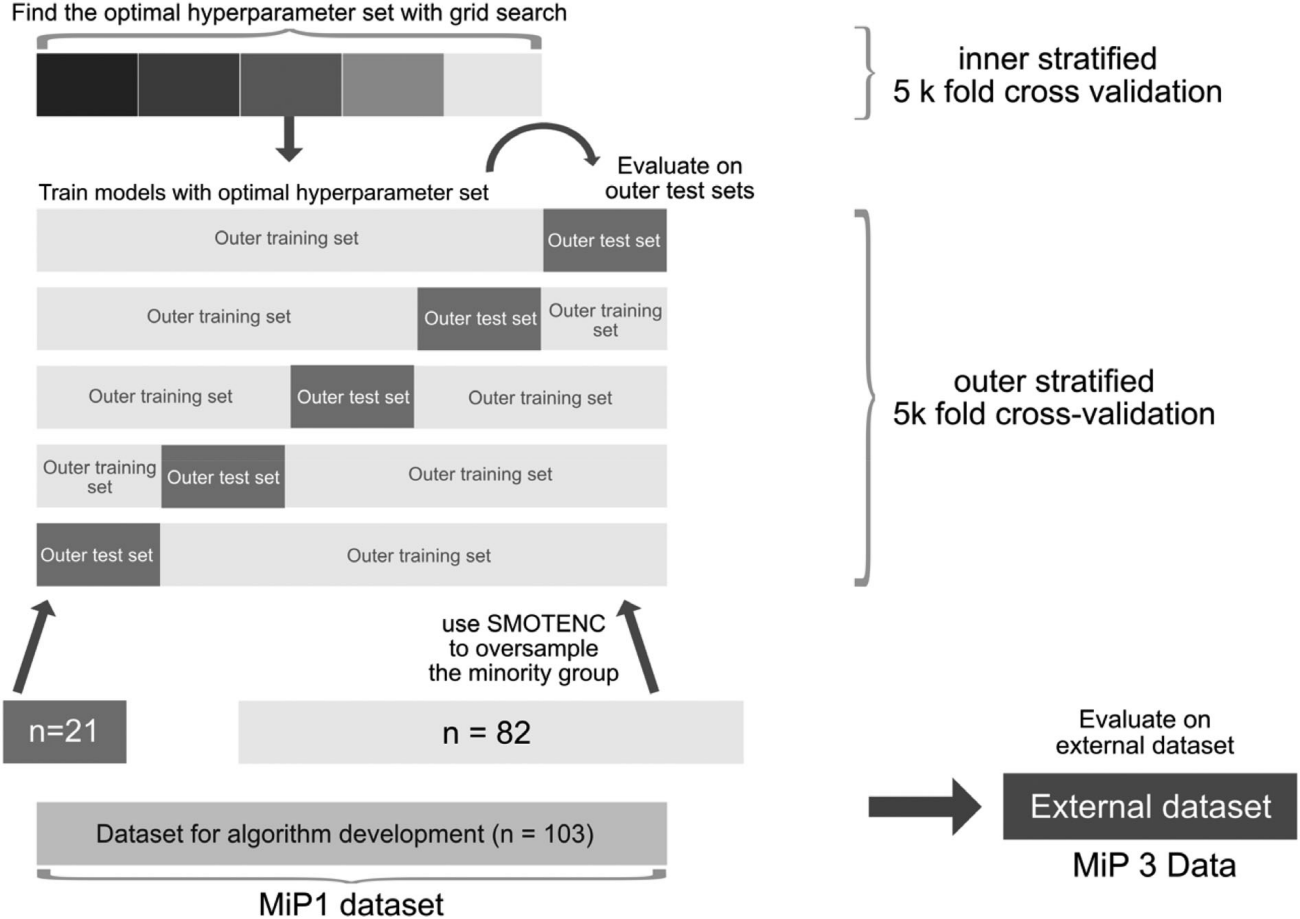
Illustration of the 5 × 5 nested cross‐validation framework employed for training the machine learning algorithms.

We trained the ML classifiers within a 5 × 5 nested cross‐validation framework. Due to class imbalance, we used the SMOTENC (Synthetic Minority Over‐sampling Technique for Nominal and Continuous) algorithm and adjusted class weights to enhance balance within the training set. This algorithm generates synthetic instances by considering the unique characteristics of categorical features, effectively mitigating class imbalance in data sets with mixed data types. We used grid‐search to determine the best parameter combination in the inner loop and tested the performance of the model in the outer loops, as indicated in Figure 1.

To evaluate the models' performances, we calculated mean Accuracy scores, as well as the minimum, maximum, mean, and standard deviation of balanced accuracies, recall, and F1 scores obtained from the five outer cross‐validation folds. Additionally, we generated AUC‐ROC curves and Calibration Curves providing a comprehensive visualization of the model evaluation (see Supplements). An external validation data set was only available for the first decision tree model, since not all parameters of the MiP 1 study were collected in the MiP 3 study.

For analysis, Python version: 3.9.16 with the packages, Pandas (version: 1.5.2), Scipy (version 1.10.0), NumPy (version: 1.24.3), Scikit‐learn (version: 1.3.2), Imbalanced‐learn (version: 1.3.2) and Matplotlib (version: 3.6.2), dtreeviz (version: 2.2.0), XGBoost (version 1.7.4) and statmodels (version: 0.13.5) were used. The code for model 1 is provided in the supplements.

## 3 RESULTS

### 3.1 Patient Population

The sample used is a subsample of the MiP 1 study, which is described in a previous paper.^16^ 163 patients were initially included. 103 (63.2%) patients for whom weight was available in week four were considered for further analysis. The sample consisted of participants of Caucasian origin, 60 (58.6%) were men. On average, the 103 study participants gained 1.7 kg (±3.23) in 4 weeks. 21 participants (20.4%) experienced a weight gain of ≥5%. Comparing the characteristics of the two groups (< and ≥5% weight gain), weight gain measured in kilogram was deemed significant after FDR correction (t[1] = −11.20 *p* = <0.01) (see Table 1).

**TABLE 1:** Demographic and clinical characteristics of the MiP1 population at baseline with weight gain of <5% versus >5% weight gain.

	<5% weight gain			≥5% weight gain			
	M	SD	*N*	M	SD	*N*	*p*
Age	41.90	14.40	82	38.24	15.21	22	
Age first psychiatric symptoms	29.10	15.72	82	27.67	14.69	22	
Number of children	0.67	1.07	82	0.62	1.16	22	
Number of WGAM	1.50	0.81	80	1.57	0.68	22	
Weight change (kg)	0.5	1.96	82	6.40	2.91	22	**<0.01**
RR systolic	129.39	17.98	80	123.95	13.87	20	
RR diastolic	82.18	9.78	80	79.30	9.44	20	
Pulse	83.26	14.92	80	80.10	13.72	20	
BMI	26.78	5.99	82	24.13	5.06	21	
Premorbid BMI	24.78	5.03	57	24.68	3.96	15	
Waist circumference	97.20	15.26	82	90.48	14.75	21	
HbA1c (%)	5.24	0.51	71	5.15	0.33	17	
Leukocytes (G/l)	6.67	1.90	81	6.68	1.60	21	
TAG (mg/dL)	142.75	96.24	77	137.33	84.10	18	
Fasting glucose (mg/dL)	90.23	16.52	70	97.11	21.68	18	
HDL (mg/dL)	54.72	18.78	76	53.47	13.68	19	
LDL (mg/dL)	117.41	40.12	76	107.33	27.35	18	
FEV 1 cognitive control	5.22	5.21	82	3.52	4.77	21	
FEV 2 disinhibition	4.22	3.99	82	5.57	4.96	21	
FEV 3 hunger	3.59	3.53	82	5.42	5.25	21	
FEV total	11.93	10.04	82	12.62	12.35	21	
FC fat	5.03	5.18	82	7.04	6.98	21	
FC sweets	7.62	7.10	82	7.04	7.92	21	
FC carbohydrates	6.43	5.68	82	6.14	5.80	21	
FC fastfood	3.86	3.72	82	3.76	3.69	21	
FC total	21.89	18.13	82	24.00	20.62	21	

	*%*		*N*	%		*N*	*p*
Gender (female)	41.46		34/82	42.86		9/21	
Diabetes	7.32		6/82	0		0/21	
Smoker	43.90		36/82	38.09		8/21	
F20 diagnosis	20.73		16/82	4.76		1/21	
Drug addiction	18.29		15/82	19.05		4/21	
Affective disorder	71.95		59/82	76.19		16/21	

*Note*: *p*‐values are FDR corrected. Highlighted values represent the top‐performing results among each algorithm for each row.

Abbreviations: BMI, body mass index; M, mean; *N*, number of patients; SD, standard deviation; TAG, triglyceride level; WGAM weight gain associated medication.

### 3.2 MiP 3 population

Compared to the MiP 1 data set, a weight gain of at least 5% only occurred in 7.89% of the cases making extensive weight gain less likely in the MiP 3 data set, which was used for external validation. A comparison of demographic and clinical characteristics of both samples is presented in Table 2.

**TABLE 2:** Demographic and clinical characteristics of the MiP 1 and the MiP 3 cohort.

	*MiP 1*			*MiP 3*			
	M	SD	*N*	M	SD	*N*	*p*
Age	41.16	14.57	103	40.73	14.50	38	
Age‐first psychiatric symptoms	28.81	15.45	103	24.68	15.47	38	
Number of WGAM	1.51	0.78	103	1.32	0.53	38	
Weight change (kg)	1.70	3.23	103	0.24	2.50	38	**0.05**
RR systolic	128.30	17.31	100	123.53	16,69	38	
RR diastolic	81.60	9.73	100	77.21	14.58	38	0.12^a^
Pulse	82.63	14.67	100	80.87	14.18	38	
BMI	26.24	5.87	103	25.07	6.49	38	
Premorbid BMI	24.76	4.80	72	22.02	4.76	33	**0.03**
Waist circumference	95.83	15.33	103	78.68	15.62	38	**<0.01**
HbA1c	5.22	0.48	88	5.32	1.39	38	
Leukocytes (G/L)	6.68	1.80	102	6.18	1.76	38	
TAG (mg/dL)	141.73	93.65	95	132.23	109.26	37	
Fasting glucose (mg/dL)	91.64	17.78	88	90.34	25.24	38	
HDL (mg/dL)	54.57	17.82	95	56.82	15.25	38	
LDL (mg/dL)	115.48	38.09	94	111.37	32.98	38	

	*%*		*N*	*%*		*N*	*p*
Gender (female)	41.42		43/103	63.16		24/38	0.12^a^
Diabetes	5.82		6/103	0		0/38	
Smoker	42.71		44/103	28.95		11/38	**0.01**
F20 diagnosis	17.48		18/103	0		0/38	**0.02**
Drug addiction	18.45		19/103	0		0/38	**<0.01**
Affective disorder	72.82		75/103	100		38/38	

*Note*: *p*‐values are FDR corrected. Highlighted values represent the top‐performing results among each algorithm for each row.

Abbreviations: BMI, body mass index; M, mean; *N*, number of patients; SD, standard deviation; TAG, triglyceride level; WGAM weight gain associated medication.

^a^ Significant before FDR‐correction.

Participants of the MiP 3 study showed a mean weight gain of 0.24 kg (±2.50) at week 4. Comparing the MiP 1 and MiP 3 population, a highly significant difference in waist circumference was found (t[1] = 5.86 *p* = <0.01), as well as the pre‐morbid BMI (t[1] = 2.73 *p* = <0.01), and the amount of weight gain within 4 weeks was significantly different (t[1] = −11.07 *p* < 0.01). Since the MiP3 data set solely consisted of depressed patients, significant differences were also found concerning patients' psychopathology (see Table 2).

#### 3.2.1 MODEL 1—Based on factors from literature

The first model, solely based on factors found in literature, yielded in a logistic regression model (Log‐Likelihood value [9] = −44.17, *p* = 0.07), with McFadden Pseudo R‐squared value of 0.15, which was not significant. Within the model only the factors BMI at admission (*p*‐value: 0.02 95% CI: 0.64–0.95 OR: 0.78) and the premorbid BMI (*p*‐value: 0.01 95% CI: 1.03–1.55 OR: 1.27) were significant.

**FIGURE 2:**
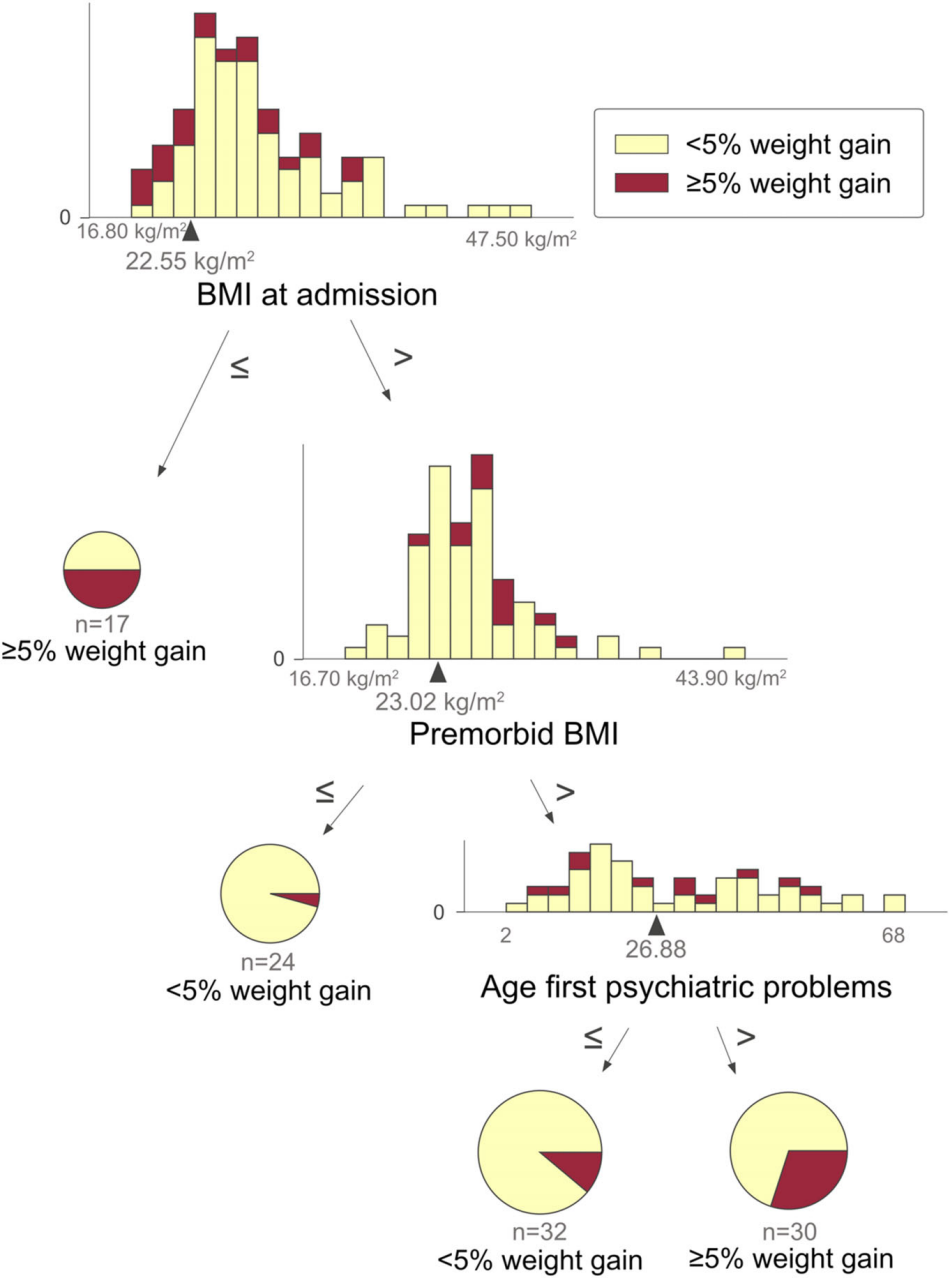
The decision tree based on factors retrieved from a literature search.

The decision tree model, as depicted in Figure 2, demonstrated a Balanced Accuracy (BAC) of 62.50% and an area under the curve (AUC) of 0.70 in its respective outer test sample. The aggregated BAC across all outer test samples was 64.03% (±1.92%). In the external validation sample, the BAC was 59.05%. Notably, the Decision Tree had a recall of 0.66 (See Table 3). According to the decision tree one would gain more than 5% of his or her weight within a week if the BMI at admission is less than or equal to 22.55 kg/m^2^.

**TABLE 3:** Comparison of performance across machine learning models on the MIP1 and the MiP3 data set.

	MiP 1				
	Dummy classifier^a^	Logistic regression	Decision tree classifier	SVM classifier	XGBoost classifier
Mean accuracy	**79.62% ± 1.77%**	65.14% ± 5.90%	63.14% ± 8.86%	72.95% ± 11.30%	70.90% ± 2.69%
Min accuracy	**76.19%**	57.14%	52.38%	52.38%	66.67%
Max accuracy	80.95%	75.00%	75.00%	**85.00%**	75.00%
Mean balanced accuracy	50.00% ± 0.00%	62.10% ± 2.34%	**64.03% ± 1.92%**	62.67% ± 12.08%	48.35% ± 4.63%
Mean precision	0.00 ± 0.00	0.32 ± 0.47	0.32 ± 0.43	**0.50 ± 0.28**	0.10 ± 0.12
Mean recall	0.00 ± 0.00	0.57 ± 0.10	**0.66 ± 0.13**	0.46 ± 0.26	0.10 ± 0.12
Mean F1 score	0.00 ± 0.00	0.31 ± 0.20	**0.42 ± 0.02**	0.40 ± 0.15	0.10 ± 0.12

	MiP 3				
	Dummy classifier^a^	Logistic regression	Decision tree classifier	SVM classifier	XGBoost classifier
Accuracy	**92.11%**	42.11%	52.63%	57.89%	68.42%
Balanced accuracy	50.00%	**68.57%**	59.05%	46.67%	37.14%
Precision	0.00	**0.12**	0.10	0.07	0.0
Recall	0.00	**1.00**	0.66	0.33	0.0
F1 score	0.00	**0.21**	0.18	0.11	0.0

*Note*: Results of model 1 (factors from literature research) when applied to MiP3 data set. Highlighted values represent the top‐performing results among each algorithm for each row.

^a^ The dummy classifier always predicts the majority class within the data sets.

If subjects have a BMI greater than 22.55 kg/m^2^ and a premorbid BMI less or equal to 23.02, the model predicts no weight gain exceeding or equal to 5% within 4 weeks. If the premorbid BMI is greater than 23.02 kg/m^2^ and the age at the onset of the first psychiatric problems is greater than 26.88, weight gain equal to or exceeding 5% is predicted. In younger individuals weight gain of <5% is predicted. The performance metrics for the Decision Tree algorithm and Logistic Regression surpassed those of the XGBoost algorithm and SVM algorithm, as indicated by BAC, precision and recall (See Table 3).

#### 3.2.2 MODEL 2

For Model 2 further laboratory values and questionnaire data were included. Calculating the Spearman correlation coefficient indicated multicollinearity for the variables FEV and FC total, leading to the removal of both sum scores. The resulting logistic regression model was significant (Log‐Likelihood value [18] = −35.77, *p* = 0.02) with a pseudo‐*R* ^2^ of 0.31. Looking at individual factors within the regression model BMI at admission (*p*‐value = 0.02 95% CI: 0.46–0.94 OR: 0.66), blood glucose (*p*‐value: 0.03 95% CI: 1.00–1.09 OR: 1.05), and Gender (*p*‐value: 0.04 95% CI: 1.04–26.6 OR: 5.28), were significant.

There was no external validation data set available for the second decision tree model (see Figure 2). The Decision Tree Classifier showed a mean accuracy of 73.67% (±9.34%). The balanced accuracy, accounting for imbalanced data sets, was 69.42% (±13.83%). The mean recall and the mean F1 score across the outer test folds were 0.62 (±0.25) and 0.48 (±0.18), respectively (see Table 4).

**TABLE 4:** Comparison of Model 2's performance across various machine learning models on the MIP1 data set.

	Evaluated learning models				
	Dummy classifier^a^	Logistic regression	Decision tree classifier	SVM classifier	XGBoost classifier
Mean accuracy	**79.62% ± 1.7** **7%**	66.05% ± 9.22%	73.67% ± 9.34%	75.71% ± 8.59%	78.62% ± 2.52%
Min accuracy	**76.19%**	55.00%	60.00%	61.90%	75.00%
Max accuracy	80.95%	80.00%	**85.71%**	**85.71%**	80.95%
Mean balanced accuracy	50.00% ± 0.00%	57.57% ± 8.28%	**69.42% ± 13.83%**	58.01% ± 10.74%	65.36% ± 12.24%
Mean precision	0.00 ± 0.00	0.31 ± 0.13	0.32 ± 0.09	**0.43 ± 0.34**	0.42 ± 0.22
Mean recall	0.00 ± 0.00	0.43 ± 0.98	**0.62 ± 0.25**	0.28 ± 0.17	0.43 ± 0.33
Mean F1 score	0.00 ± 0.00	0.35 ± 0.11	**0.48 ± 0.18**	0.32 ± 0.21	0.39 ± 0.21

*Note*: Results from outer folds in the nested cross‐validation framework.

Abbreviation: SVM, support vector machine.

^a^ The dummy classifier always predicts the majority class within the data sets.

**FIGURE 3:**
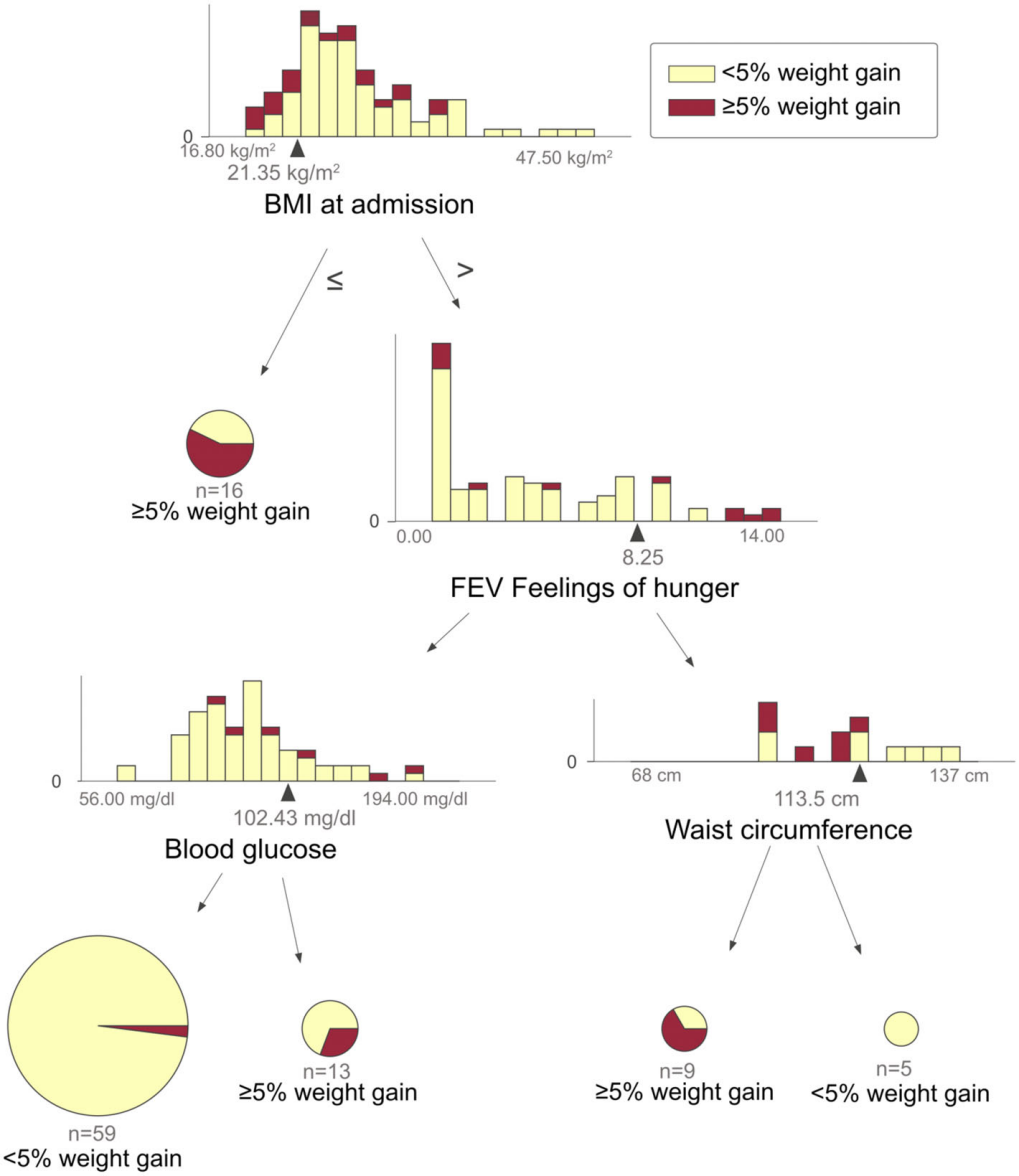
The decision tree based on the addition of further factors to the ones from literature.

Model 2 predicts a weight gain of ≥5% within 4 weeks if the individual has a BMI at admission below or equal to 21.35 kg/m^2^. Additionally, if the BMI at admission is above this threshold, the FEV feelings of hunger scale is below 8.25, and fasting glucose is below 102.43 mg/dL, the model predicts no weight gain of 5% or more within 4 weeks. However, if the glucose level exceeds 102.43 mg/dL, the model predicts a weight gain of more than 5% of the body weight at admission. If the FEV feelings of hunger scale exceeds the value of 8.25 in a specific patient, and their waist circumference is below 113.5 cm, the model predicts a weight gain of more than 5% of the body weight at admission. However, no such weight gain is predicted if the waist circumference is above 113.5 cm (Figure 3).

## 4 DISCUSSION

The aim of this study was to uncover modifiable predictors of PIWG, as well as to identify patients at‐risk for PIWG. This allows future at‐risk patients to take pre‐emptive measures and receive targeted interventions to prevent weight gain in patients taking psychotropic medication. About 20% of the patients enrolled in this study experienced a weight gain of at least 5% within the first 4 weeks of their drug treatment, which is in accordance with previous data in the literature.^12^, ^13^ The logistic regression model of the factors found in literature, which had a *p*‐value of 0.07, suggests that the overall model was not significant. Notably, within the logistic regression the premorbid BMI and the BMI admission were significant, both factors are well‐known predictors for PIWG. In the context of the decision tree model based on literature factors, here named Model 1, the age at the onset of the first psychiatric problems also contributed to the model's predictions, where, in addition to a high premorbid BMI and low BMI at admission, the onset of disease above the age of 27 was associated with a higher risk for PIWG. Despite differences in weight gain prevalence and psychiatric diagnoses, the first decision tree model demonstrates generalizability among a diverse cohort of psychiatric patients newly prescribed medications associated with PIWG.

Complex models, such as SVM and XGBoost, may face challenges in calibration. In smaller data sets (*N* = 103), the limited instances hinder the learning of nuances within the underlying distribution, resulting in high variability in calibration across different folds during the cross‐validation process. Well‐calibrated models are more reliable in real‐world settings, particularly in applications where accurate probability estimates are crucial for decision‐making. Notably, both the decision tree model and the Regression Model exhibit the best calibration, although there is room for improvement (see Data S1).

Considering the significant impact of PIWG on patients, it is advantageous to prioritize a high level of sensitivity in diagnostic or predictive models. Patients were monitored for 4 weeks, possibly insufficient for those experiencing delayed PIWG, potentially leading to increased false negative rates. During phases of acute psychiatric symptomatology, patients may initially lose weight before experiencing weight gain as a positive healing response, which should be considered when monitoring PIWG. Therefore the premorbid BMI should be taken into account in the assessment of PIWG, especially in young adults with a slim physique. One possible limitation in this regard is the use of a questionnaire to measure past weight, introducing the potential for unreliable results due to recall bias with patients either over‐ or underestimating their previous weight.

The setup for Model 2 included questionnaires on dietary habits and eating behavior, as well as fasting glucose. This model demonstrated statistical significance, as indicated by a Log‐Likelihood value of −35.77 and a *p*‐value of 0.02. The McFadden pseudo‐*R* ^2^ value of 0.31 indicates good model fit, since the pseudo‐*R* ^2^ metric doesn't equal to the conventional *R* ^2^ metric.^42^ Compared to the factors found in the literature the second regression model outperformed model 1 regarding the *p*‐value as well as the pseudo *R* ^2^ metric.

Within the model, higher BMI at admission was significantly associated with a lower probability to gain at least 5% of weight within 4 weeks as indicated by the OR with a decrease of an associated risk of 33.86%. An increased blood glucose was significantly associated with a 3% increased risk of gaining ≥5% of body weight.

In the corresponding decision tree model, the mean BAC value improved in all models except the logistic regression and the dummy classifier compared to Model 1, indicating that additional factors provide crucial prognostic information. Both logistic regression and the decision tree model identified BMI at admission and fasting glucose levels, highlighting their significance.

Due to the absence of the FC questionnaire and the presence of another FEV questionnaire in the MiP 3 study, external validation was unavailable for the second regression model and its corresponding decision tree.

This raises the question of whether Model 2 is as generalizable as Model 1. However, it also suggests that focusing on lifestyle and behavioral factors may be important when evaluating someone's risk of weight gain in general. To date, research has not extensively examined these modifiable risk factors, and there is limited available data. In Model 2, eating behavior emerged as an important factor in predicting whether someone will experience PIWG, in addition to BMI at admission and blood glucose levels. Study participants with a BMI > 21.35 kg/m^2^ had a higher risk for gaining ≥5% of weight if they had a high level of hunger and a low waist circumference or higher levels of blood glucose. The lower waist circumference again fits the predictive value of a lower BMI, despite the number of patients in this arm of the decision tree being very small. High levels of blood glucose can be altered by lifestyle measures and medication.

The FEV subscale “hunger” represents a modifiable variable that can be addressed in a preventive therapy program, as our work group was already able to show in the MiP 2 study.^43^ The metabolic dysfunction during treatment usually goes along with feelings of ravenousness,^44^ partly driven by appetite‐increasing side effects of weight gain associated drugs, underpinned by biological changes in appetite control.^45^ In healthy women the state of hunger has a huge impact on food craving, which is further modulated by emotional state^46^ – a connection that might be even more pronounced in psychiatric patients. Regarding the operationalization of eating behavior by the FEV it should be noted that there is a high correlation between the disinhibition and hunger scale.^36^ The sensation of hunger may be reduced by pharmacological interventions,^47^ where the combination of pharmacological and non‐pharmacological interventions is the most effective approach.^9^, ^48^

Identifying and addressing these factors in at‐risk PIWG patients before weight gain can prevent additional suffering, as well as increased mortality, and morbidity.^8^ However, it must be emphasized that not all patients who did not gain ≥5% of weight were metabolically healthy. In the total MiP 1 sample, 30.1% of patients were overweight, 17.2% were obese, and 26.9% and 22.4% met the criteria for metabolic syndrome.^16^

## 5 CONCLUSION

Since not every patient treated with weight gain associated medication is gaining weight and resources should be used economically, a filter is needed to identify patients who will experience relevant weight gain with all its possible long‐term metabolic sequelae. We propose a functioning model predicting PIWG. It offers valuable decision criteria that clinicians can readily utilize. These criteria are based on easily collectible and cost‐saving factors, making them highly practical. In addition modifiable risk factors can be addressed therapeutically. Consequently, the findings of our study suggest further data collection on a larger scale to further refine the identification of individuals at risk and to further validate our findings.

## FUNDING INFORMATION

This study was not funded by any specific grant or external funding source. The research described in this paper was primarily conducted through the contributions of the authors' personal resources and institutional support. Julia Eder, Lisa Pfeiffer, and Peter Falkai have received funding and support from the German Research Foundation through their participation in the Graduate College POKAL (DFG‐GrK 2621). Further Julia Eder received funding from the “University Foundation” (Stiftungsmittel der Universität) at LMU for general medical research purposes, specifically through Foundations in support of the Medical Faculty. Maria S. Simon received funding through the Föfole (“Förderung für Forschung und Lehre”) program at LMU. While these funds did not directly support the current study, they have contributed to the broader research efforts of the authors and have facilitated their ongoing scholarly pursuits.

## CONFLICT OF INTEREST STATEMENT

Richard Musil has received financial research support from the EU (H2020 No. 754740), and served as PI in clinical trials from Abide Therapeutics, Böhringer‐Ingelheim, Emalex Biosciences, Lundbeck GmbH, Nuvelution TS Pharma Inc., Oryzon, Otsuka Pharmaceuticals and Therapix Biosciences. Peter Falkai received research support/honoraria for lectures or advisory activities from: Boehringer‐Ingelheim, Janssen, Lundbeck, Otsuka, Recordati, and Richter. All other authors declare that they have no conflicts of interest.

### PEER REVIEW

The peer review history for this article is available at https://www.webofscience.com/api/gateway/wos/peer-review/10.1111/acps.13684.

## Supporting information


**Data S1:** Supporting Information.

**Data S2:** Supporting Information.

## Data Availability

The data used in this research concerns anonymized patient level data, suitable for use by researchers, after permission from the members of the MIP team and the POKAL group. However, due to binding legislation and institutional policy sharing of these data to third parties is not possible. The MiP 1 data shown in this paper was also used for a previous publication of the MIP team.^49^
