# Supplementary material for: Who is at risk for weight gain after weight‐gain associated treatment with antipsychotics, antidepressants, and mood stabilizers: A machine learning approach
Source: Acta Psychiatr Scand. 2024 Apr 1;151(3):231–44. doi: 10.1111/acps.13684 (PMC11787916; doi:10.1111/acps.13684)
Supplement: Supplementary file 1 — Data S1: Supporting Information. [file ACPS-151-231-s002.pdf]

Who is at risk for weight gain after weight-gain associated treatment with antipsychotics, antidepressants, and mood stabilisers: A machine learning approach.

Commented code to replicate model 1

Import Libraries

In [1]:

```
#import necessary libraries
import pandas as pd
import numpy as np
import sklearn
import matplotlib.pyplot as plt
import matplotlib as mpl
import scipy as sp
import seaborn as sns
import sys
from sklearn.impute import KNNImputer
from sklearn.tree import DecisionTreeClassifier
from sklearn.metrics import confusion_matrix
from sklearn.metrics import precision_recall_curve, auc, roc_curve
from sklearn.calibration import calibration_curve
from sklearn.preprocessing import RobustScaler
from imblearn.over_sampling import SMOTENC
from sklearn.svm import SVC
from sklearn.model_selection import StratifiedKFold,GridSearchCV
from sklearn.metrics import balanced_accuracy_score, precision_score, recall_score
from sklearn.metrics import f1_score, accuracy_score, classification_report
from sklearn.linear_model import LogisticRegression
from sklearn.dummy import DummyClassifier
from xgboost import XGBClassifier
from sklearn.metrics import make_scorer
```

```python
from itertools import product
import warnings
import xgboost as xgb
import statsmodels
import IPython
from IPython import display
from sklearn.svm import SVC
from sklearn.linear_model import LogisticRegression
from sklearn.dummy import DummyClassifier
from xgboost import XGBClassifier
from sklearn.metrics import make_scorer
from imblearn.over_sampling import SMOTENC
import statsmodels.api as sm
import dtreeviz
from itertools import product
import warnings
```

Import Data

MiP 1 Data

In [2]:
```python
raw_data = pd.read_excel('MIP2.xlsx', sheet_name=0, header=0, engine='openpyxl')

start = raw_data.columns.get_loc("Clozapin0")
end = raw_data.columns.get_loc("Zopiclon4")

for x in range(len(raw_data.dtypes)):
    if raw_data.dtypes[x] == object:
        new_vals = pd.to_numeric(raw_data.iloc[:, x], errors='coerce')
        raw_data[raw_data.columns[x]] = new_vals.fillna(0)

raw_data2 = pd.read_excel('for_Publikation/MIP1_vollst.xlsx') #needed to calculate BMI. Ana_14 is size in cm.
nu = raw_data2.loc[:, ["ID", "Ana_14"]].copy()
raw_data = raw_data.merge(nu, how='left', left_on='ID', right_on='ID').copy()
```

Preprocess MiP 1 data

In [3]:
```
# In the column 'Weight_before_mental_illness' ('Gewicht_vor_psych_Erkrank'), some subjects have reported 0 here
#
# These values are set to "Nan".
# Similar cases apply for LDL, HbA1c, HDL, TAG etc.
raw_data['Gewicht_vor_psych_Erkrank'] = raw_data['Gewicht_vor_psych_Erkrank'].replace(0, np.nan) # premorbid weig
raw_data['pre_BMI']=raw_data['Gewicht_vor_psych_Erkrank']/(raw_data['Ana_14']**2).copy() #calculate premorbid BMI
raw_data['Labor_LDL'] = raw_data['Labor_LDL'].replace(0, np.nan) # values with 0 are replaced with Nan
raw_data['Labor_HbA1c'] = raw_data['Labor_HbA1c'].replace(0, np.nan)
raw_data['TG_HDL'] = raw_data['TG_HDL'].replace(0, np.nan)
raw_data['Labor_Glucose'] = raw_data['Labor_Glucose'].replace(0, np.nan)
raw_data['Labor_Leuko'] = raw_data['Labor_Leuko'].replace(0, np.nan)
raw_data['Labor_TAG'] = raw_data['Labor_TAG'].replace(0, np.nan)
raw_data['weight_gain_5_percent'] = raw_data['weight_gain_5_percent'].replace(2, 0).copy()
raw_data['education'] = raw_data['Abitur'].replace(1, 3).copy()
raw_data['education'] = raw_data['education']+raw_data['MittlereReife'].replace(1, 2).copy()
raw_data['education'] = raw_data['education']+raw_data['Hauptschulabschluss'].replace(1, 1).copy()
raw_data['F20']=raw_data.pschotic_disorders+raw_data.schizoaffective_disorders.copy()
raw_data['F20']=raw_data['F20'].replace(2, 1).copy()
raw_data['Labor_HDL'] = raw_data['Labor_HDL'].replace(0, np.nan)
raw_data['Alter_erste_psychAuffälligkeit'] = raw_data['Alter_erste_psychAuffälligkeit'].replace(0, np.nan)
```

Through a literature review, various factors were identified that influence weight gain under psychotropic drugs. Specifically, these are ['BMI_0', 'pre_BMI', 'Labor_TAG', 'Gender', 'Age_first_mental_symptom', 'Age', 'F20', 'drug_addiction', 'Smoking_yes_no', 'Labor_HDL']

In [4]:
```
list_col2=['BMI_0','pre_BMI','Labor_TAG','Geschlecht', 'Alter_erste_psychAuffälligkeit','Alter',
            'F20','drug_addiction','Rauchen_ja_nein','Labor_HDL']
```

Get MiP 3 data

In [5]:
```
## MiP 3 Data
validierung_POK=pd.read_excel('validierung_pokal.xlsx')

validierung_POK['education'] = (
```

```
    validierung_POK['Abitur ja/nein'].replace(1, 3) +
    validierung_POK['Mittlere Reife ja/nein'].replace(1, 2) +
    validierung_POK['Hauptschulabschluss ja/nein'].replace(1, 1)
).copy()

validierung_POK['F20'] = np.zeros((len(validierung_POK['education']), 1))
validierung_POK['drug_addiction'] = np.zeros((len(validierung_POK['education']), 1))

validierung_POK['BMI_0'] = (
    validierung_POK['Koerpergewicht_0'] /
    ((validierung_POK['Körpergröße (cm)'] / 100) ** 2)
)

validierung_POK['pre_BMI'] = (
    validierung_POK['prämorbides-Gewicht(kg)'] /
    ((validierung_POK['Körpergröße (cm)'] / 100) ** 2)
)
#rename columns if necessary
validierung_POK.rename(columns = {'Labor_TAG (mg/dl)':'Labor_TAG'}, inplace = True)
validierung_POK.rename(columns = {'Lab_Glucose':'Labor_Glucose'}, inplace = True)
validierung_POK.rename(columns = {'Lab_HDL (mg/dl)':'Labor_HDL'}, inplace = True)
validierung_POK.rename(columns = {'Lab_LDL (mg/dl) ':'Labor_LDL'}, inplace = True)

validierung_POK.rename(columns = {'Diabetes ja/nein':'Diabetes_ja_nein_Medis_berücksichtigt'}, inplace = True)
validierung_POK.rename(columns = {'Lab_Leukozyten (G/l)':'Labor_Leuko'}, inplace = True)
validierung_POK.rename(columns = {'Lebt alleine':'Lebt_alleine'}, inplace = True)
validierung_POK.rename(columns = {'aktuell berufstätig ja/nein':'aktuell_berufstätig'}, inplace = True)

validierung_POK['TG_HDL']=validierung_POK['Labor_TAG'] / validierung_POK['Labor_HDL']

validierung_POK['Ana_14']=validierung_POK['Körpergröße (cm)']/100

x13= validierung_POK.loc[:,list_col2];
bool_target2=validierung_POK["weight_gain_5_percent"]>0.0;
```

In [6]:

```
x2=raw_data.loc[:,list_col2]
x2.BMI_0=round(x2.BMI_0,1)
x2.Labor_HDL=round(x2.Labor_HDL,1)
x2.Alter=round(x2.Alter,1)
```

```
x2.Alter_erste_psychAuffälligkeit=round(x2.Alter_erste_psychAuffälligkeit,1)
x2.pre_BMI=round(x2.pre_BMI,1)
x2.Labor_TAG=round(x2.Labor_TAG,1)
```

Remove datasets with too many missings

In [7]:
```
##delete rows with too many missings
# Calculate the threshold for missing values
threshold = 0.80 * len(x2.columns)

# Filter rows in x2 with more than 20% missing values
x2 = x2.dropna(thresh=threshold, axis=0)

# Use the index of x2 to filter raw_data
raw_data = raw_data.loc[x2.index]
bool_target=raw_data["weight_gain_5_percent"]>0.0


# Print the shapes of the resulting dataframes
print("Rows     : ", x2.shape[0])
print("Columns : ", x2.shape[1])
```

```
Rows     :  103
Columns :  10
```

KNN Imputation

In [8]:
```
#impute missing values with knn imputation
imputer = KNNImputer(n_neighbors=5, weights="uniform")
X=pd.DataFrame(imputer.fit_transform(x2),columns=[list_col2])
y =  bool_target
```

MAIN CODE

In [9]:
```
warnings.filterwarnings("ignore", category=RuntimeWarning)
```

```python
from sklearn.exceptions import UndefinedMetricWarning
def warn(*args, **kwargs):
    pass

warnings.warn = warn
warnings.filterwarnings("ignore", category=UndefinedMetricWarning)

np.random.seed(42)


class ModelOptimizer:
    def __init__(self, trials=100, outer_k=5, inner_k=5, class_weights='balanced'):
        self.trials = trials
        self.outer_k = outer_k
        self.inner_k = inner_k
        self.class_weights = class_weights
        self.best_model= None;
        self.inner_acc=-1

    def _get_base_model(self, model_type, params=None):
        if model_type == 'DecisionTree':
            return DecisionTreeClassifier(
                criterion='gini',max_features=5, min_impurity_decrease=0.007,
                splitter='best', class_weight=self.class_weights,
                max_leaf_nodes=6,
                ccp_alpha=0.0165,random_state=11
            )
        elif model_type == 'SVM':
            return SVC(probability=True, class_weight=self.class_weights,random_state=0)
        elif model_type == 'LogisticRegression':
            return LogisticRegression(class_weight=self.class_weights,random_state=0)
        elif model_type == 'Dummy':
            return DummyClassifier(strategy='constant', constant=0)
        elif model_type == 'XGBoost':
            return XGBClassifier(n_estimators=2, max_depth=2, learning_rate=1, objective='binary:logistic',
                                 random_state=0)

    def _calculate_and_plot_curves(self, y_true, y_pred_prob, ax, curve_type='prc'):
        if curve_type == 'prc':
```

```python
            precision, recall, _ = precision_recall_curve(y_true, y_pred_prob)
            ax.plot(recall, precision, marker='o', label='Precision-Recall Curve')
            no_skill = 0.2 #that's how often weight gain > 5% occurs
            ax.plot([0, 1], [no_skill, no_skill], linestyle='--', color='gray')

            ax.set_xlabel('Recall')
            ax.set_ylabel('Precision')
            ax.set_title('Precision-Recall Curve')
            ax.legend()
        elif curve_type == 'roc':
            fpr, tpr, _ = roc_curve(y_true, y_pred_prob)
            roc_auc = auc(fpr, tpr)
            ax.plot(fpr, tpr, marker='o', label=f'ROC Curve (AUC = {roc_auc:.2f})')

            ax.set_xlabel('False Positive Rate')
            ax.set_ylabel('True Positive Rate')
            ax.set_title('AUC-ROC Curve')
            ax.legend()

    def optimize_model(self, X, y, model_type='DecisionTree', param_grid=None, use_smote=False):
        np.random.seed(42)

        imputer = KNNImputer(n_neighbors=5, weights="uniform")
        X = pd.DataFrame(imputer.fit_transform(X), columns=[list_col2])
        y = bool_target


        outer_cv = StratifiedKFold(n_splits=self.outer_k, shuffle=True, random_state=18)
        if param_grid is None:
                param_grid = {}
        # Initialize the GridSearchCV for the inner loop
        inner_cv = StratifiedKFold(n_splits=self.inner_k, shuffle=True, random_state=42)
        model = self._get_base_model(model_type)

        grid_search = GridSearchCV(estimator=model, param_grid=param_grid, cv=inner_cv,
                        scoring=['balanced_accuracy', 'precision','recall'], refit='balanced_accuracy')

        outer_accuracies = []
        outer_precisions = []
```

```
        outer_recalls = []
        outer_f1_scores = []
        outer_specificities = []
        outer_balanced_accuracies = []
        fig, axs = plt.subplots(1, 3, figsize=(15, 5))

        for train_idx, test_idx in outer_cv.split(X, y):
                X_train, X_test = X.iloc[train_idx], X.iloc[test_idx]
                y_train, y_test = y.iloc[train_idx], y.iloc[test_idx]


                if use_smote:
                    # Apply SMOTENC to the training data
                    smote_nc = SMOTENC(categorical_features=[3,6,7],
                                       random_state=42,sampling_strategy=0.4)




                    X_resampled, y_resampled = smote_nc.fit_resample(X_train, y_train)


                    X_train = pd.DataFrame(X_resampled, columns=X_train.columns)
                    y_train = pd.Series(y_resampled)

                grid_search.fit(X_train, y_train)
                best_model = grid_search.best_estimator_
                self.best_model=best_model
                y_pred = best_model.predict(X_test)

                y_pred_inner = best_model.predict(X_train)
                balanced_accuracy = balanced_accuracy_score(y_train, y_pred_inner)

                accuracy = best_model.score(X_test, y_test)
                precision = precision_score(y_test, y_pred, average='binary',pos_label=True)
                recall = recall_score(y_test,y_pred, average='binary',pos_label=True)
                f1 = f1_score(y_test, y_pred, average='binary',pos_label=True)

                tn, fp, fn, tp = confusion_matrix(y_test, y_pred).ravel()
                specificity = tn / (tn + fp)
```

```python
                balanced_accuracy = balanced_accuracy_score(y_test, y_pred)


                outer_accuracies.append(accuracy)
                outer_precisions.append(precision)
                outer_recalls.append(recall)
                outer_f1_scores.append(f1)
                outer_specificities.append(specificity)
                outer_balanced_accuracies.append(balanced_accuracy)

                y_prob = best_model.predict_proba(X_test)[:, 1]

                self._calculate_and_plot_curves(y_test, y_prob, axs[0], curve_type='prc')

                # Plot AUC-ROC Curve
                self._calculate_and_plot_curves(y_test, y_prob, axs[1], curve_type='roc')

                fop, mpv = calibration_curve(y_test, y_prob, n_bins=10)
                # Plot Calibration Curve
                axs[2].plot(mpv, fop, marker='o', label=f'Fold {len(outer_balanced_accuracies)}')



        axs[2].set_xlabel("Mean Predicted Probability")
        axs[2].set_ylabel("Fraction of Positives")
        axs[2].plot([0, 1], [0, 1], linestyle='--', color='gray')
        axs[2].set_title("Calibration Curve")
        axs[1].plot([0, 1], [0, 1], linestyle='--', color='gray')

        axs[2].legend()

        fig.suptitle(f"{model_type}", fontsize=20)

        plt.tight_layout()
        plt.show()

        mean_accuracy = np.mean(outer_accuracies)
        std_accuracy = np.std(outer_accuracies)
```

```
        mean_precision = np.mean(outer_precisions)
        std_precision = np.std(outer_precisions)

        mean_recall = np.mean(outer_recalls)
        std_recall = np.std(outer_recalls)

        mean_f1 = np.mean(outer_f1_scores)
        std_f1 = np.std(outer_f1_scores)

        mean_specificity = np.mean(outer_specificities)
        std_specificity = np.std(outer_specificities)
        if model_type == 'LogisticRegression':
                    X_with_intercept = sm.add_constant(X_train)

                    # Fit the logistic regression model
                    logit_model = sm.Logit(y_train, X_with_intercept)
                    result = logit_model.fit()

                    # Display the summary with coefficients and p-values
                    print(result.summary())
        mean_balanced_accuracy = np.mean(outer_balanced_accuracies)
        std_balanced_accuracy = np.std(outer_balanced_accuracies)
        print("Nested Cross-Validation Mean Accuracy: {:.2f}% ± {:.2f}%".format(100 * mean_accuracy,
                                                                                 100 * std_accuracy))
        print("Nested Cross-Validation Min Accuracy: {:.2f}%".format(100 * np.min(outer_accuracies)))
        print("Nested Cross-Validation Max Accuracy: {:.2f}%".format(100 * np.max(outer_accuracies)))

        print("Nested Cross-Validation Mean Precision: {:.2f}% ± {:.2f}%".format(100 * mean_precision,
                                                                                  100 * std_precision))
        print("Nested Cross-Validation Mean Recall: {:.2f}% ± {:.2f}%".format(100 * mean_recall, 100 * std_recal
        print("Nested Cross-Validation Mean F1 Score: {:.2f}% ± {:.2f}%".format(100 * mean_f1, 100 * std_f1))
        print("Nested Cross-Validation Mean Specificity: {:.2f}% ± {:.2f}%".format(100 * mean_specificity,
                                                                                    100 * std_specificity))
        print("Nested Cross-Validation Mean BAC: {:.2f}% ± {:.2f}%".format(100 * mean_balanced_accuracy,
                                                                            100 * std_balanced_accuracy))
```

In [10]:
```
dt_param_grid = {
    "max_depth": [2,3,4],
    "max_features": [ 3,5, None],
```

```
    "min_samples_split":[ 5],
    "min_samples_leaf":[ 5,12],
    "max_leaf_nodes":[  8,10]
}

# Set parameters for SVM optimization
svm_param_grid = {
    "C": [0.1, 1, 10],
    "kernel": ["linear","rbf"],
    "gamma": ["scale", "auto"]
}

# Set parameters for Logistic Regression optimization
lr_param_grid = {
    "C": [0.1, 1, 10],
    "penalty": ["l1", None]
}
# Set parameters for XGBoost optimization

xgb_param_grid = {
    'learning_rate': [0.01, 0.1, 0.2],
    'n_estimators': [20,50],
    'max_depth': [2,3],
    'gamma': [0, 0.1, 0.2],
}
```

In [11]:

```
def report (optimizer,x_train,y_train,x_holdout,y_holdout):
    print('Train_Test:')

    print('Classification report:')
    print(classification_report(y_train, optimizer.predict(imputer.fit_transform(x_train ))))

    print('Confusion matrix:')
    print(confusion_matrix(y_train, optimizer.predict(imputer.fit_transform(x_train ))))
    y_pred_v=optimizer.predict(imputer.fit_transform(x_holdout))

    # Print the classification report and confusion matrix
    print('Performance on MiP 3 data')
    print('External Validation:')
```

```
    print('Classification report:')
    print(classification_report(y_holdout, y_pred_v))

    print('Confusion matrix:')
    print(confusion_matrix(y_holdout, y_pred_v))

    print('Accuracy')
    balanced_acc = accuracy_score(y_holdout, y_pred_v)
    print(balanced_acc)
    print('BAC')
    balanced_acc = balanced_accuracy_score(y_holdout, y_pred_v)
    print(balanced_acc)

    print('F1')
    f1 = f1_score(y_holdout, y_pred_v, average='binary',pos_label=True)
    print(f1)
    print('Precision')
    pre = precision_score(y_holdout, y_pred_v, average='binary',pos_label=True)
    print(pre)
    print('Recall')
    re = recall_score(y_holdout, y_pred_v, average='binary',pos_label=True)
    print(re)
```

Logistic Regression Results

In [12]:

```
model_optimizer = ModelOptimizer()

scaler = RobustScaler()
X_scaled = pd.DataFrame(scaler.fit_transform(X),columns=X.columns)

X13_scaled = pd.DataFrame(scaler.fit_transform(x13),columns=x13.columns)

print ('Logistic regression')
model_optimizer.optimize_model( X_scaled, y, model_type='LogisticRegression',
                               param_grid=lr_param_grid,use_smote=True)
print(model_optimizer.best_model)
report(model_optimizer.best_model,X_scaled,bool_target ,X13_scaled ,bool_target2 )
```

Logistic regression

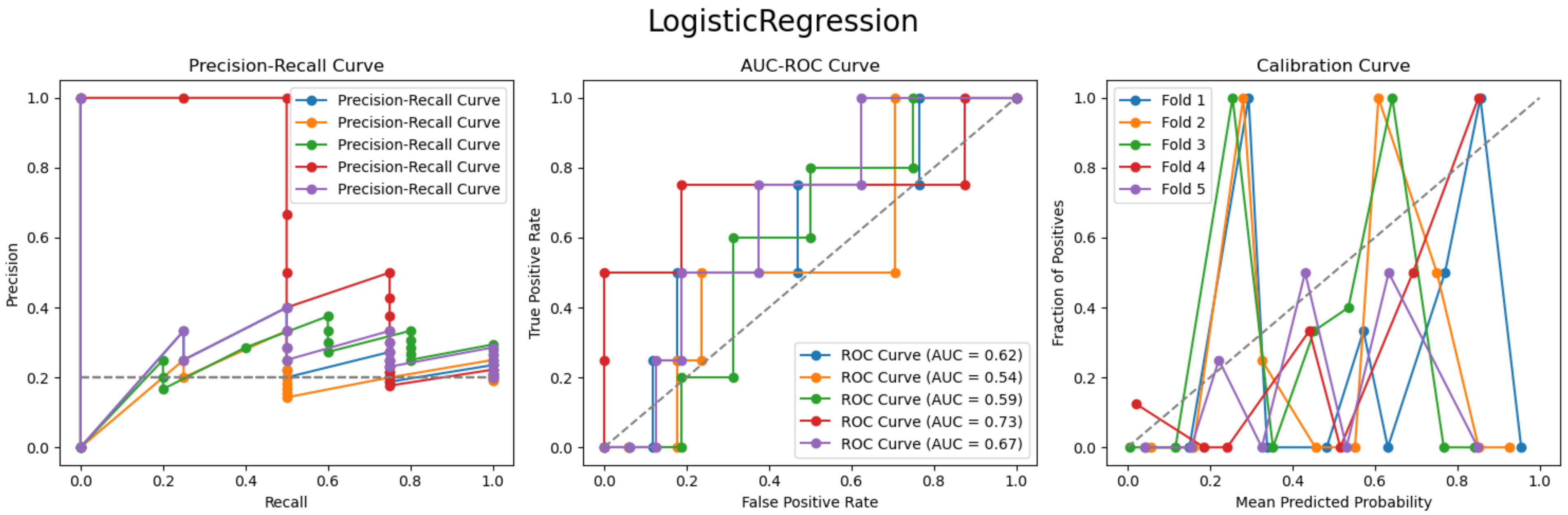


```
Optimization terminated successfully.
         Current function value: 0.501862
         Iterations 7
                           Logit Regression Results
================================================================================
Dep. Variable:     weight_gain_5_percent   No. Observations:                   92
Model:                             Logit   Df Residuals:                       81
Method:                              MLE   Df Model:                           10
Date:                   Wed, 28 Feb 2024   Pseudo R-squ.:                  0.1571
Time:                           16:48:45   Log-Likelihood:                -46.171
converged:                          True   LL-Null:                       -54.777
Covariance Type:               nonrobust   LLR p-value:                   0.06982
======================================================================================================
                                        coef    std err          z      P>|z|      [0.025      0.975]
------------------------------------------------------------------------------------------------------
const                                -0.1111      0.451     -0.246      0.806      -0.996       0.774
('BMI_0',)                           -1.1875      0.629     -1.889      0.059      -2.419       0.044
('pre_BMI',)                          0.6706      0.505      1.327      0.185      -0.320       1.661
('Labor_TAG',)                       -0.1076      0.280     -0.384      0.701      -0.657       0.442
('Geschlecht',)                       0.0409      0.659      0.062      0.950      -1.251       1.333
```

```
('Alter_erste_psychAuffälligkeit',)     0.4811      0.645      0.746      0.456      -0.784       1.746
('Alter',)                             -0.4374      0.632     -0.692      0.489      -1.676       0.801
('F20',)                               -1.7077      1.146     -1.490      0.136      -3.954       0.538
('drug_addiction',)                    -1.0543      0.934     -1.129      0.259      -2.885       0.776
('Rauchen_ja_nein',)                   -1.2360      0.596     -2.073      0.038      -2.405      -0.067
('Labor_HDL',)                         -0.4007      0.549     -0.729      0.466      -1.478       0.676
=========================================================================================================
Nested Cross-Validation Mean Accuracy: 65.14% ± 5.90%
Nested Cross-Validation Min Accuracy: 57.14%
Nested Cross-Validation Max Accuracy: 75.00%
Nested Cross-Validation Mean Precision: 31.55% ± 4.70%
Nested Cross-Validation Mean Recall: 57.00% ± 9.80%
Nested Cross-Validation Mean F1 Score: 40.01% ± 3.30%
Nested Cross-Validation Mean Specificity: 67.21% ± 9.35%
Nested Cross-Validation Mean BAC: 62.10% ± 2.34%
LogisticRegression(C=0.1, class_weight='balanced', penalty=None, random_state=0)
Train_Test:
Classification report:
              precision    recall  f1-score   support

       False       0.87      0.66      0.75        82
        True       0.32      0.62      0.42        21

    accuracy                           0.65       103
   macro avg       0.59      0.64      0.58       103
weighted avg       0.76      0.65      0.68       103

Confusion matrix:
[[54 28]
 [ 8 13]]
Performance on MiP 3 data
External Validation:
Classification report:
              precision    recall  f1-score   support

       False       1.00      0.37      0.54        35
        True       0.12      1.00      0.21         3

    accuracy                           0.42        38
```

```
   macro avg       0.56      0.69      0.38        38
weighted avg       0.93      0.42      0.52        38

Confusion matrix:
[[13 22]
 [ 0  3]]
Accuracy
0.42105263157894735
BAC
0.6857142857142857
F1
0.21428571428571425
Precision
0.12
Recall
1.0
```

XGBoost Results

In [13]:

```
print ('XGBoost')
model_optimizerxgboost = ModelOptimizer()
model_optimizerxgboost.optimize_model( X_scaled, y, model_type='XGBoost', param_grid=xgb_param_grid,use_smote=Tru
print(model_optimizerxgboost.best_model)
report(model_optimizerxgboost.best_model,X_scaled,bool_target,X13_scaled ,bool_target2  )
```

```
XGBoost
```

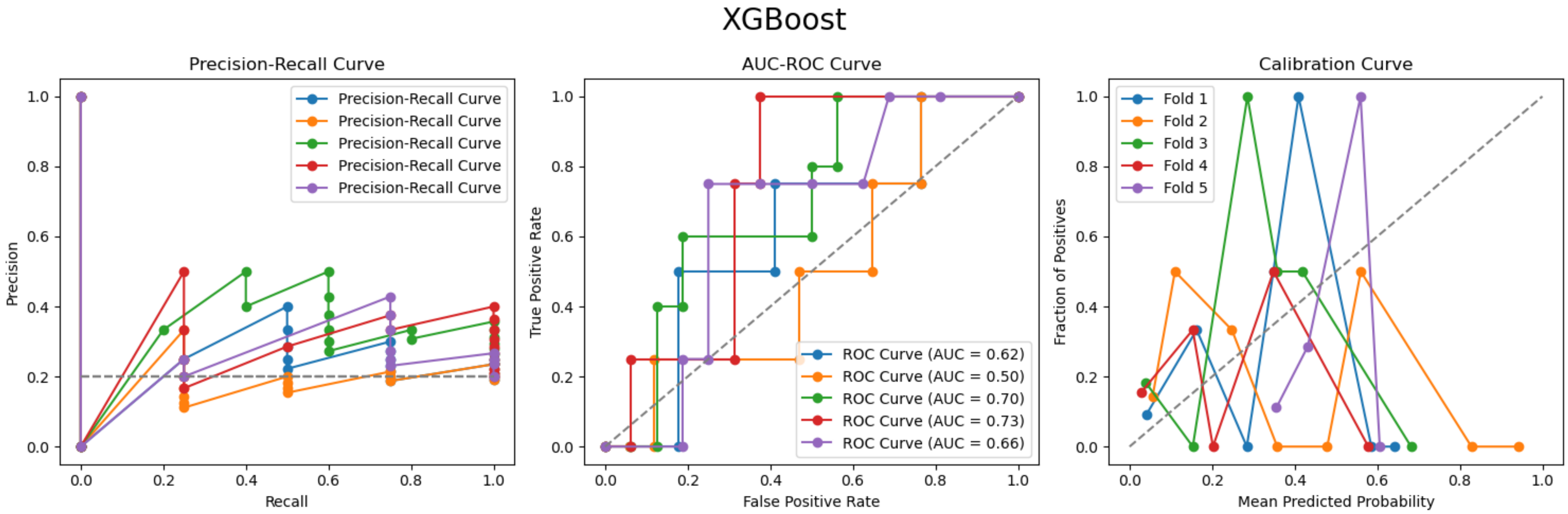


```
Nested Cross-Validation Mean Accuracy: 70.90% ± 2.69%
Nested Cross-Validation Min Accuracy: 66.67%
Nested Cross-Validation Max Accuracy: 75.00%
Nested Cross-Validation Mean Precision: 10.00% ± 12.25%
Nested Cross-Validation Mean Recall: 10.00% ± 12.25%
Nested Cross-Validation Mean F1 Score: 10.00% ± 12.25%
Nested Cross-Validation Mean Specificity: 86.69% ± 5.78%
Nested Cross-Validation Mean BAC: 48.35% ± 4.63%
XGBClassifier(base_score=None, booster=None, callbacks=None,
              colsample_bylevel=None, colsample_bynode=None,
              colsample_bytree=None, early_stopping_rounds=None,
              enable_categorical=False, eval_metric=None, feature_types=None,
              gamma=0, gpu_id=None, grow_policy=None, importance_type=None,
              interaction_constraints=None, learning_rate=0.01, max_bin=None,
              max_cat_threshold=None, max_cat_to_onehot=None,
              max_delta_step=None, max_depth=3, max_leaves=None,
              min_child_weight=None, missing=nan, monotone_constraints=None,
              n_estimators=50, n_jobs=None, num_parallel_tree=None,
              predictor=None, random_state=0, ...)
Train_Test:
Classification report:
```

```
              precision    recall  f1-score   support

       False       0.87      0.94      0.90        82
        True       0.64      0.43      0.51        21

    accuracy                           0.83       103
   macro avg       0.75      0.68      0.71       103
weighted avg       0.82      0.83      0.82       103

Confusion matrix:
[[77  5]
 [12  9]]
Performance on MiP 3 data
External Validation:
Classification report:
              precision    recall  f1-score   support

       False       0.90      0.74      0.81        35
        True       0.00      0.00      0.00         3

    accuracy                           0.68        38
   macro avg       0.45      0.37      0.41        38
weighted avg       0.83      0.68      0.75        38

Confusion matrix:
[[26  9]
 [ 3  0]]
Accuracy
0.6842105263157895
BAC
0.37142857142857144
F1
0.0
Precision
0.0
Recall
0.0
```

Dummy Predictor Results

In [14]:
```
print ('Dummy')
model_optimizerdu = ModelOptimizer()
model_optimizerdu.optimize_model( X_scaled, y, model_type='Dummy')
print(model_optimizerdu.best_model)
report(model_optimizerdu.best_model,X_scaled,bool_target ,X13_scaled ,bool_target2 )
```

```
Dummy
```

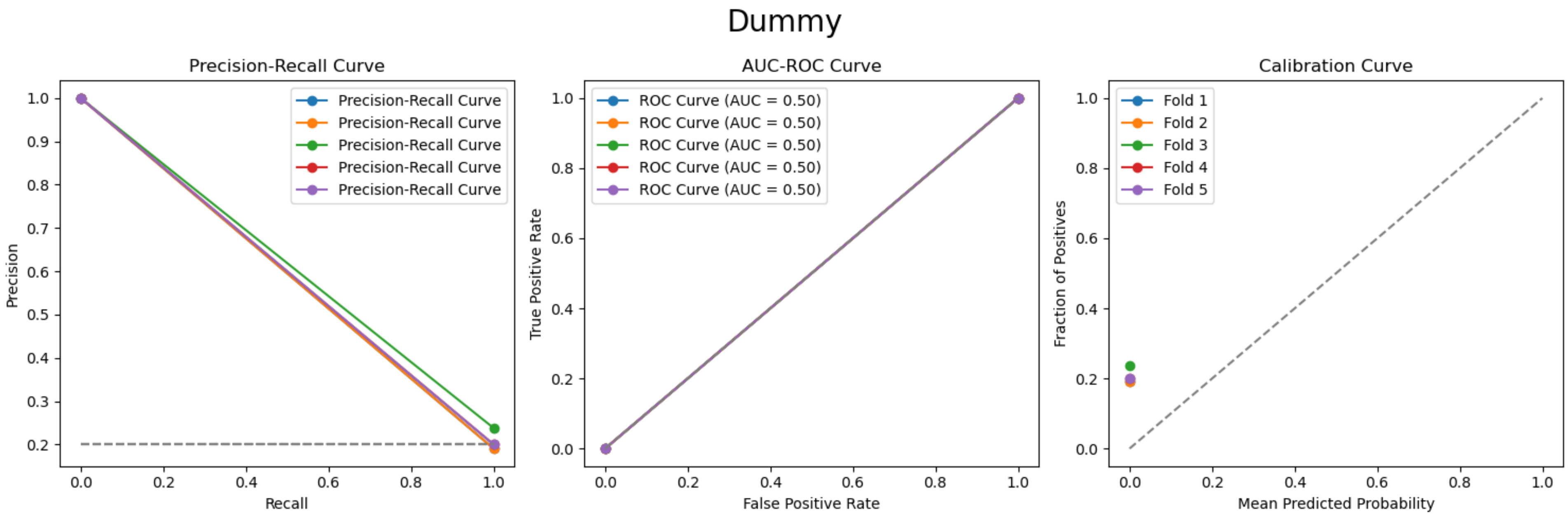


```
Nested Cross-Validation Mean Accuracy: 79.62% ± 1.77%
Nested Cross-Validation Min Accuracy: 76.19%
Nested Cross-Validation Max Accuracy: 80.95%
Nested Cross-Validation Mean Precision: 0.00% ± 0.00%
Nested Cross-Validation Mean Recall: 0.00% ± 0.00%
Nested Cross-Validation Mean F1 Score: 0.00% ± 0.00%
Nested Cross-Validation Mean Specificity: 100.00% ± 0.00%
Nested Cross-Validation Mean BAC: 50.00% ± 0.00%
DummyClassifier(constant=0, strategy='constant')
Train_Test:
Classification report:
              precision    recall  f1-score   support
```

```
       False       0.80      1.00      0.89        82
        True       0.00      0.00      0.00        21

    accuracy                           0.80       103
   macro avg       0.40      0.50      0.44       103
weighted avg       0.63      0.80      0.71       103

Confusion matrix:
[[82  0]
 [21  0]]
Performance on MiP 3 data
External Validation:
Classification report:
              precision    recall  f1-score   support

       False       0.92      1.00      0.96        35
        True       0.00      0.00      0.00         3

    accuracy                           0.92        38
   macro avg       0.46      0.50      0.48        38
weighted avg       0.85      0.92      0.88        38

Confusion matrix:
[[35  0]
 [ 3  0]]
Accuracy
0.9210526315789473
BAC
0.5
F1
0.0
Precision
0.0
Recall
0.0
```

SVM Results

In [15]:
```
print ('SVM')
model_optimizersvm = ModelOptimizer()
model_optimizersvm.optimize_model( X_scaled, y, model_type='SVM', param_grid=svm_param_grid,use_smote=True)
print(model_optimizersvm.best_model)
print('Performance on MiP 3 data')
report(model_optimizersvm.best_model,X_scaled,bool_target ,X13_scaled ,bool_target2  )
```

SVM

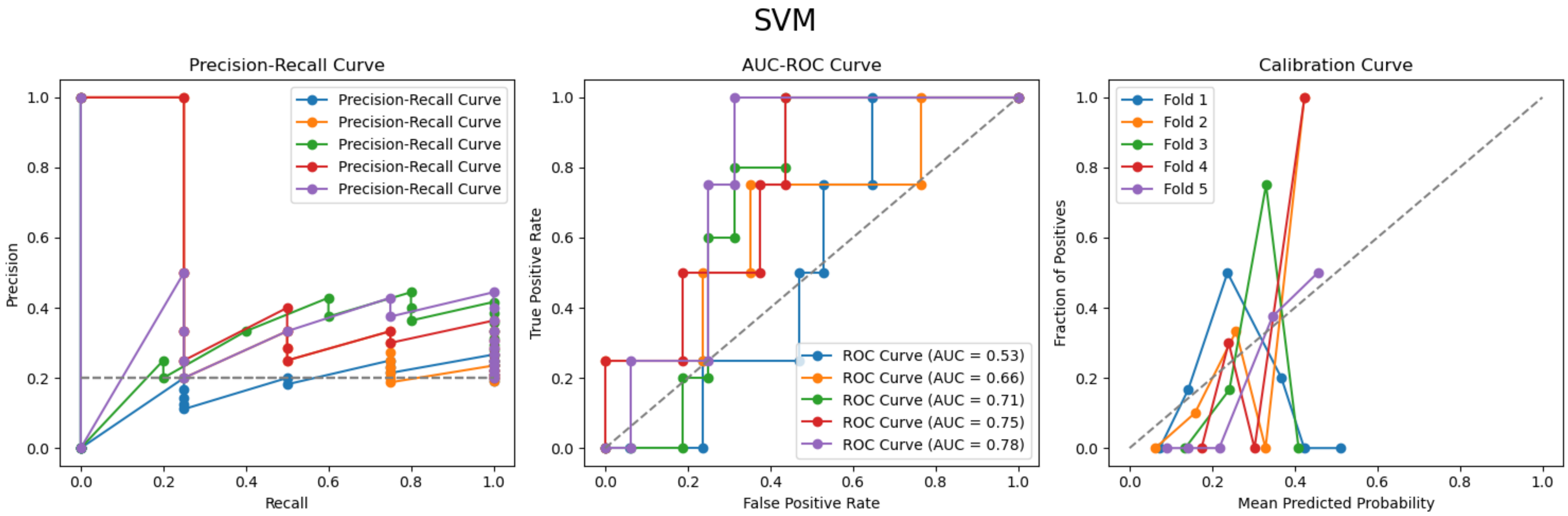


```
Nested Cross-Validation Mean Accuracy: 72.95% ± 11.30%
Nested Cross-Validation Min Accuracy: 52.38%
Nested Cross-Validation Max Accuracy: 85.00%
Nested Cross-Validation Mean Precision: 49.96% ± 28.24%
Nested Cross-Validation Mean Recall: 46.00% ± 25.77%
Nested Cross-Validation Mean F1 Score: 40.34% ± 14.79%
Nested Cross-Validation Mean Specificity: 79.34% ± 15.47%
Nested Cross-Validation Mean BAC: 62.67% ± 12.08%
SVC(C=10, class_weight='balanced', probability=True, random_state=0)
Performance on MiP 3 data
Train_Test:
Classification report:
              precision    recall  f1-score   support
```

```
       False       0.99      0.90      0.94        82
        True       0.71      0.95      0.82        21

    accuracy                           0.91       103
   macro avg       0.85      0.93      0.88       103
weighted avg       0.93      0.91      0.92       103

Confusion matrix:
[[74  8]
 [ 1 20]]
Performance on MiP 3 data
External Validation:
Classification report:
              precision    recall  f1-score   support

       False       0.91      0.60      0.72        35
        True       0.07      0.33      0.11         3

    accuracy                           0.58        38
   macro avg       0.49      0.47      0.42        38
weighted avg       0.85      0.58      0.68        38

Confusion matrix:
[[21 14]
 [ 2  1]]
Accuracy
0.5789473684210527
BAC
0.4666666666666667
F1
0.1111111111111111
Precision
0.06666666666666667
Recall
0.3333333333333333
```

Decision Tree Results

In [16]:
```python
print ('Decision Tree')
model_optimizerdt = ModelOptimizer()
model_optimizerdt.optimize_model( X, y, model_type='DecisionTree', param_grid=dt_param_grid,use_smote=True)
print(model_optimizerdt.best_model)
print('Performance on MiP 3 data')

report(model_optimizerdt.best_model,X,y,x13,bool_target2)
```

```
Decision Tree
```

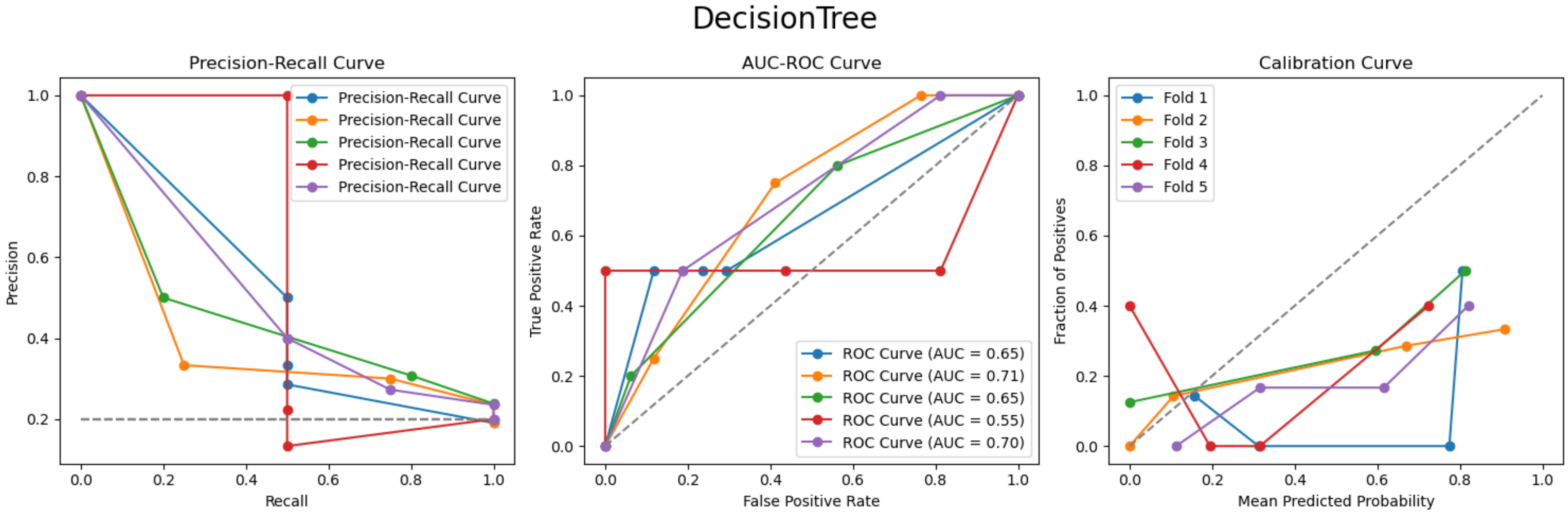


```
Nested Cross-Validation Mean Accuracy: 63.14% ± 8.86%
Nested Cross-Validation Min Accuracy: 52.38%
Nested Cross-Validation Max Accuracy: 75.00%
Nested Cross-Validation Mean Precision: 32.28% ± 4.32%
Nested Cross-Validation Mean Recall: 66.00% ± 13.19%
Nested Cross-Validation Mean F1 Score: 42.35% ± 2.00%
Nested Cross-Validation Mean Specificity: 62.06% ± 14.61%
Nested Cross-Validation Mean BAC: 64.03% ± 1.92%
DecisionTreeClassifier(ccp_alpha=0.0165, class_weight='balanced', max_depth=3,
                       max_leaf_nodes=8, min_impurity_decrease=0.007,
                       min_samples_leaf=12, min_samples_split=5,
                       random_state=11)
```

```
Performance on MiP 3 data
Train_Test:
Classification report:
              precision    recall  f1-score   support

       False       0.89      0.61      0.72        82
        True       0.32      0.71      0.44        21

    accuracy                           0.63       103
   macro avg       0.61      0.66      0.58       103
weighted avg       0.78      0.63      0.67       103

Confusion matrix:
[[50 32]
 [ 6 15]]
Performance on MiP 3 data
External Validation:
Classification report:
              precision    recall  f1-score   support

       False       0.95      0.51      0.67        35
        True       0.11      0.67      0.18         3

    accuracy                           0.53        38
   macro avg       0.53      0.59      0.42        38
weighted avg       0.88      0.53      0.63        38

Confusion matrix:
[[18 17]
 [ 1  2]]
Accuracy
0.5263157894736842
BAC
0.5904761904761904
F1
0.18181818181818182
Precision
0.10526315789473684
Recall
```

0.6666666666666666

In [17]:
```
viz = dtreeviz.model(model_optimizerdt.best_model, X, y,
                     target_name="target",
                     feature_names=X.columns,
                     class_names=['<5% weight gain', '>5% weight gain'])




v = viz.view()     # render as SVG into internal object
v
```

Out[17]:

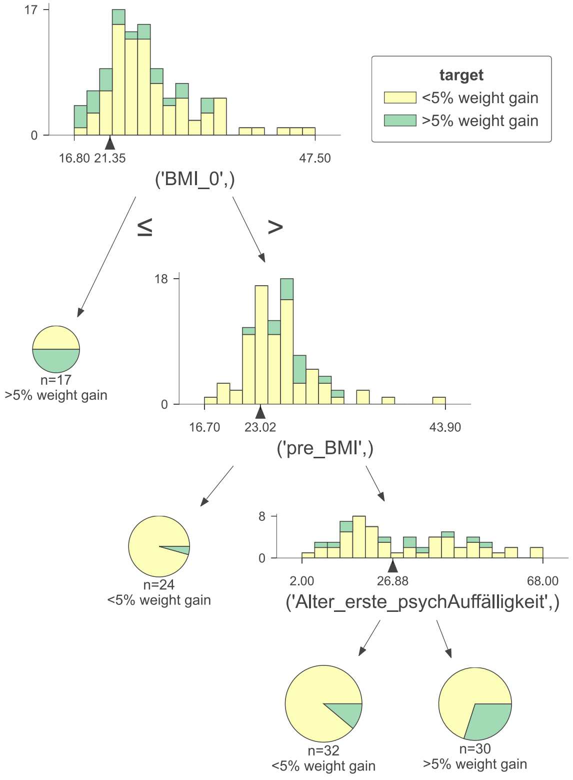

In [ ]:
