# Supplementary material for: Who is at risk for weight gain after weight‐gain associated treatment with antipsychotics, antidepressants, and mood stabilizers: A machine learning approach
Source: Acta Psychiatr Scand. 2024 Apr 1;151(3):231–44. doi: 10.1111/acps.13684 (PMC11787916; doi:10.1111/acps.13684)
Supplement: Supplementary file 2 — Data S2: Supporting Information. [file ACPS-151-231-s001.docx]

Figure a

*Comparison of Precision-Recall curves, AUC-ROC curves and Calibration curves for different Classifiers consisting of the factors from literature*


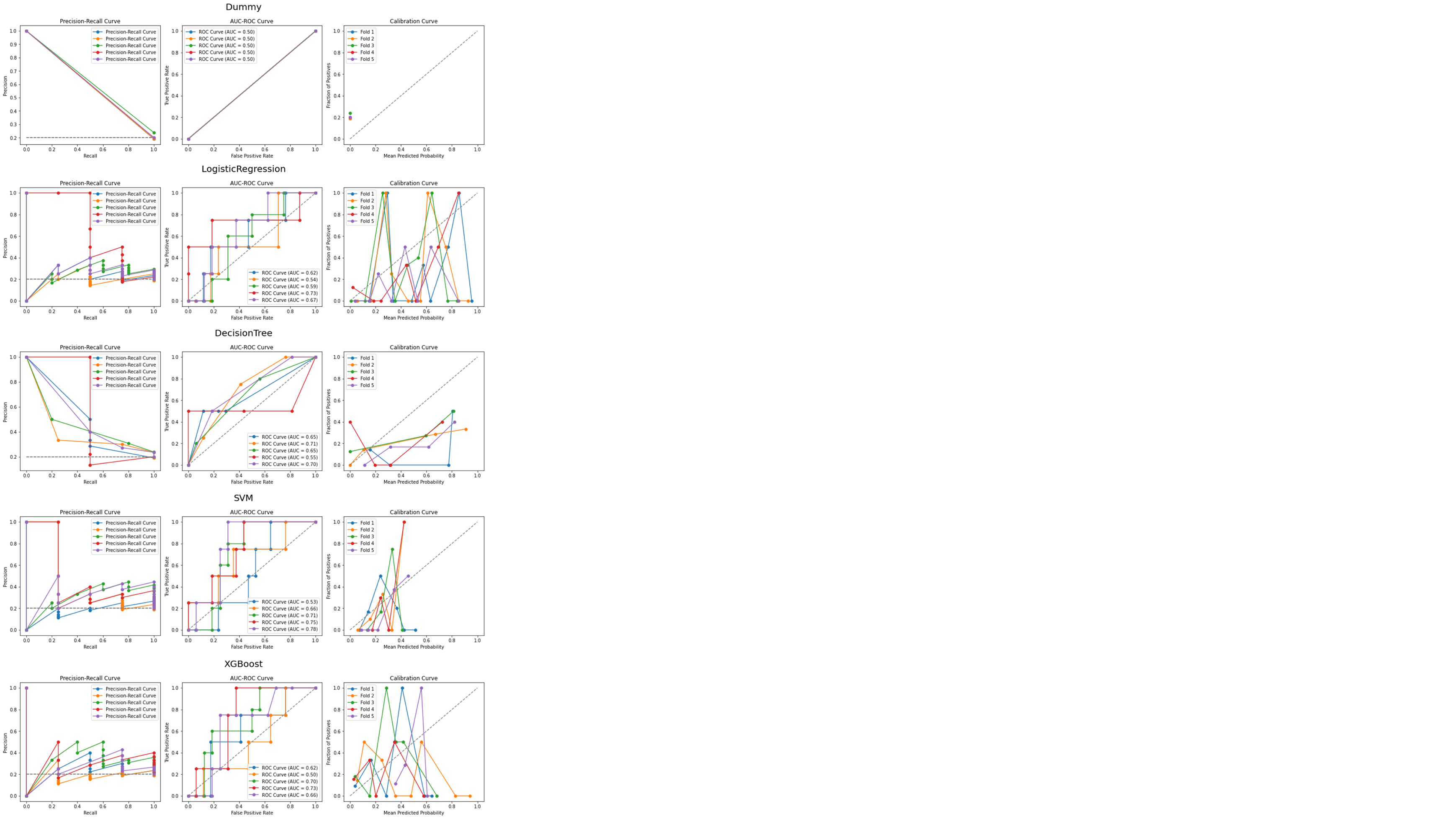


Notes. ROC receiver operater characeristic; AUC area under the curve

Figure b

*Comparison of Precision-Recall curves, AUC-ROC curves, and Calibration curves for different Classifiers, consisting of the extended feature set.*
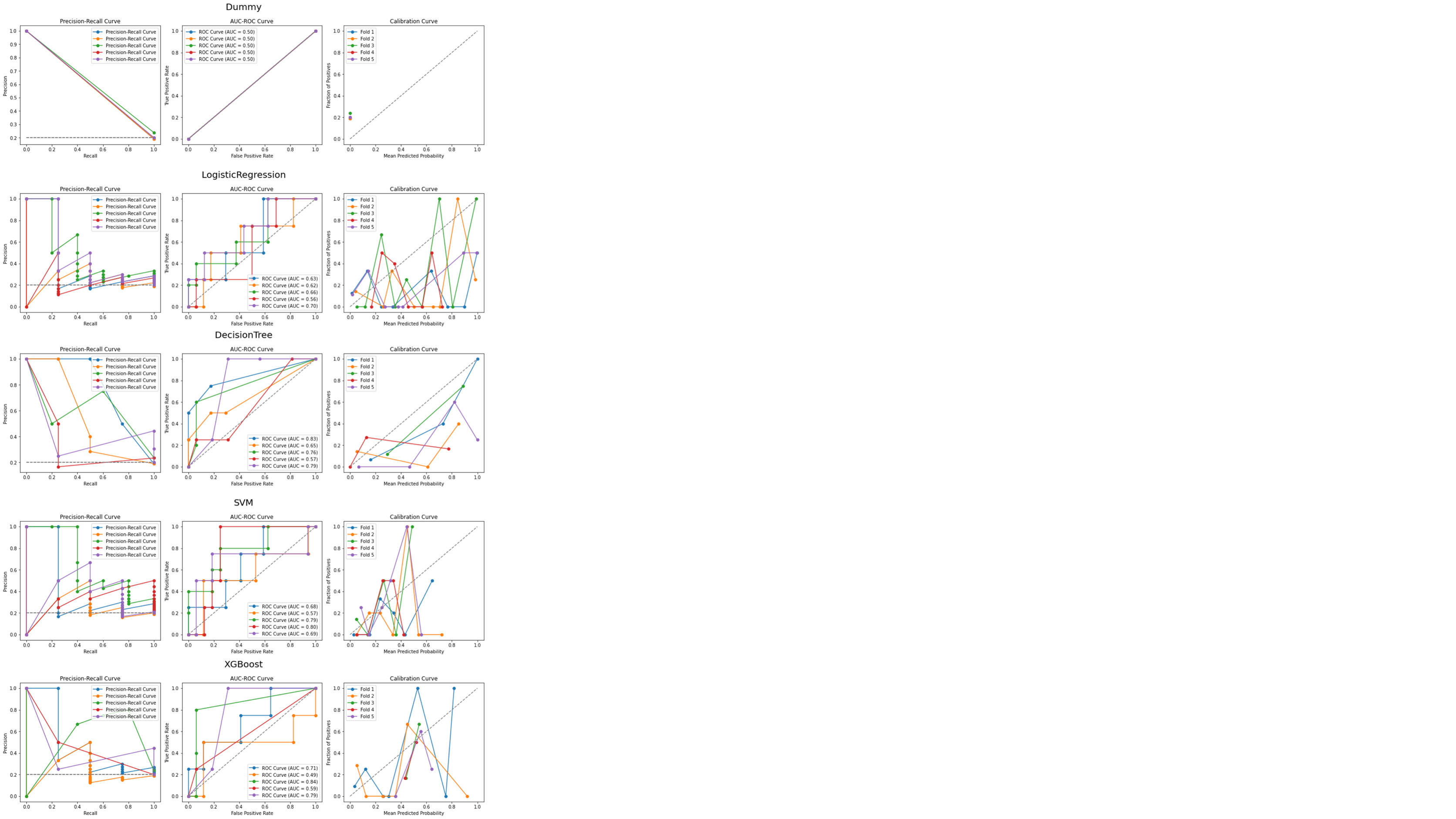


Notes. ROC receiver operater characeristic; AUC area under the curve

Hyperparameter Decision Tree Model 1:

﻿DecisionTreeClassifier(ccp_alpha=0.0165, class_weight='balanced', max_depth=3,

max_leaf_nodes=8, min_impurity_decrease=0.007,

min_samples_leaf=12, min_samples_split=5,

random_state=11)

Hyperparameter Decision Tree Model2:

﻿DecisionTreeClassifier(ccp_alpha=0.038, class_weight='balanced', max_depth=4,

max_features=26, max_leaf_nodes=8,

min_impurity_decrease=0.037, min_samples_leaf=5,

min_samples_split=4, random_state=11)
